# Altered actin isoforms expression and enhanced airway responsiveness in asthma: the crucial role of β-cytoplasmic actin

**DOI:** 10.3389/fphys.2025.1627443

**Published:** 2025-09-05

**Authors:** Marisol Alvarez-González, Angélica Flores-Flores, Verónica Carbajal-Salinas, Blanca Bazán-Perkins

**Affiliations:** ^1^ Laboratorio de Inmunofarmacología, Instituto Nacional de Enfermedades Respiratorias Ismael Cosio Villegas, Mexico City, Mexico; ^2^ Tecnologico de Monterrey, Escuela de Medicina y Ciencias de la Salud, Mexico City, Mexico

**Keywords:** contraction, hyperresponsiveness, smooth muscle, actin, asthma, airways

## Abstract

Airway hyperresponsiveness, caused by excessive contraction of airway smooth muscle, is a characteristic of asthma involving multiple proteins, including various isoforms of actin and myosin. While α-smooth muscle actin (ACTA2) is linked to hypercontractility, the roles of other isoforms are unclear. Our study investigated the expression of proteins involved in airway smooth muscle contraction and their relation to AHR in an allergic asthma model. Male guinea pigs were sensitized and challenged with ovalbumin, with controls receiving saline. We measured broncho-obstruction and AHR using plethysmography. Protein expression in bronchial and tracheal smooth muscle was analyzed through immunohistochemistry, with proteins identified using electrophoresis and MALDI/TOF-TOF mass spectrometry. In the asthma model, guinea pigs exhibited AHR. The expression of ACTA2, β-cytoplasmic actin (ACTB), and myosin light chains (MYL9) increased, while γ-cytoplasmic actin 1 (ACTG1) was reduced in the bronchial smooth muscle compared to controls. ACTB and ACTA2 expression levels were correlated with AHR, and ACTB was associated with ACTA2, MYL9, and filamin A (FLNA), and inversely with ACTG1. ACTA2 and MYL9 levels showed an inverse association with ACTG1, and the expression levels of FLNA and MYL9 were correlated. Reduced ACTG1 expression was linked to greater AHR. Proteomic analysis confirmed these proteins in guinea pig tracheal smooth muscle, although expression changes differed from the bronchus, except for ACTB, which increased in the asthma model. Our data suggest that increased ACTA2 and ACTB, along with reduced ACTG1, are related to AHR in guinea pigs. MYL9 and FLNA emerge as potential regulators of actin dynamics.

## 1 Introduction

Asthma, affecting approximately 300 million people worldwide, is a heterogeneous disease characterized by variable symptoms and fluctuating expiratory airflow limitations. This condition presents several phenotypes and endotypes, with allergic asthma being the most extensively studied. A significant pathophysiological aspect of the disease is airway hyperresponsiveness (AHR) (Global Initiative for Asthma, 2024), which is an exaggerated and rapid contraction of the smooth muscle in the airways, primarily within the bronchial tree (Gao et al., 2024).

The mechanism of airway smooth muscle (ASM) contraction is mediated by various signaling pathways, involving the interaction and regulation of multiple proteins, including essential components of the cytoskeleton such as actin and myosin filaments (Álvarez-Santos et al., 2020). Myosin heavy chains (MYH11) are crucial for smooth muscle contraction as they slide along the actin filaments, while myosin light chains (MYL9) regulate myosin activity by phosphorylating the 20 kDa myosin light chain at serine residue 19, thereby facilitating the interaction (Wang et al., 2021; Sun et al., 2020).

In terms of actin, human cells express six isoforms: α-cardiac, α-skeletal, α-SMA (ACTA2), γ-enteric, β-cytoplasmic actin (ACTB), and γ-cytoplasmic actin 1 (ACTG1). ACTA2 is involved in smooth muscle contraction, while ACTB and ACTG1 play key roles in cytoskeletal reorganization, structural integrity, motility, and cell adhesion (Suresh and Diaz, 2021; Tang, 2015). Regardless of the actin isoform, the organization and stabilization of the cytoskeleton are mediated by actin-binding proteins such as transgelin (TAGLN) and filamin A (FLNA). These proteins act as scaffolds for various transmembrane signaling complexes, which are essential for detecting and transmitting mechanical forces generated by actin-myosin interactions, as well as processes related to the maintenance of cell morphology, adhesion to the extracellular matrix, and cell migration (Iwamoto et al., 2018; Razinia et al., 2012).

MYH11, MYL9, ACTA2, and TAGLN are involved in various pathologies, including the excessive contraction observed in ASM in asthma (Woodman et al., 2008; Yu et al., 2022). Notably, it has been demonstrated that ACTA2 plays a crucial role in human bronchial smooth muscle contraction and the development of AHR (Slats et al., 2008). In contrast, there are no studies directly linking ACTB and ACTG1 to AHR. Paradoxically, the increase in actin filament formation in response to stimulation and the subsequent increase in ASM contraction does not appear to be solely the result of nucleation mediated by ACTA2; ACTB and probably ACTG1 are also involved (Zhang et al., 2005).

However, studies conducted on bovine tracheal tissue and human ASM cultures have shown no changes in ACTB levels when used as a molecular weight control marker or as a constitutive gene in various molecular techniques (Tran et al., 2006; Dekkers et al., 2007). Despite this, ACTG1 exhibits a 99% genetic similarity with ACTB, and some authors suggest that these proteins share cellular functions and could substitute specific functions interchangeably (Konig, 2013). Therefore, the regulation of not only ACTA2 but also ACTG1 may be involved in the increased contraction of ASM in asthma. In this context, the present article investigates the role of contractile proteins, particularly actins, in ASM and their relationship with increased contraction and AHR in an allergic asthma model in guinea pigs.

## 2 Materials and methods

### 2.1 Experimental model

In general, both female and male guinea pigs can be used in allergic asthma models. However, during the development of our experimental model in both sexes, we observed considerable variability in the responses to the antigen. In females, young individuals (≈450 g) did not develop AHR, whereas adult females (>550 g) consistently did (unpublished data). This finding is consistent with previous reports by Regal et al. (2007). To avoid this age-related variability in AHR, and considering that the model requires the use of young guinea pigs, only male guinea pigs were included in the present study.

Male outbred guinea pigs (HsdPoc: DH strain), weighing between 0.35 and 0.4 kg, were obtained from Harlan Mexico City, Mexico. The animals were housed under controlled laboratory conditions, including a conventional humidity of 50%–70%, temperature, controlled light/dark cycles (12/12 h), filtered air at 21 °C ± 1 °C, and sterilized bedding. All experiments were conducted following the guidelines established in the Mexican Official Standards NOM-062-ZOO-1999 and NOM-087-ECOL-SSA1-2002. All animal procedures were conducted in accordance with protocols approved by the Scientific and Bioethics Committee of the Instituto Nacional de Enfermedades Respiratorias (Approval number: B2416; IRB organization information: IORG0003948).

### 2.2 Allergic asthma model

**FIGURE 1:**
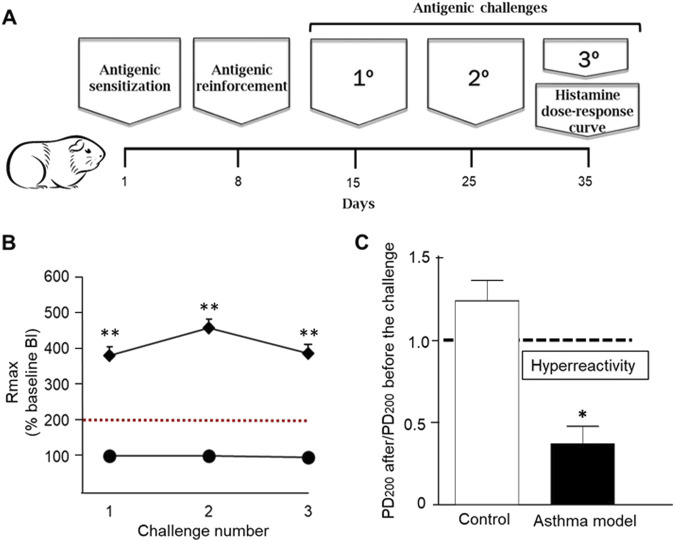
Asthma model in guinea pigs. **(A)** Day 1: Antigen sensitization with ovalbumin (OVA) and aluminum hydroxide via subcutaneous and intraperitoneal administration. Day 8: Antigen reinforcement using nebulized OVA. Days 15, 25, and 35: Antigen challenges with nebulized OVA. Day 35: Evaluation of airway reactivity through dose-response curves to increasing concentrations of histamine. **(B)** Maximum broncho-obstructive index (BI) response (Rmax) observed after antigenic challenge in sensitized guinea pigs (rhomboids) and saline-treated control groups (circles). **(C)** Airway hyperreactivity to histamine: Difference in the average 200% provocative dose (PD_200_) value before antigenic exposure compared to the PD_200_ value after antigenic exposure in response to increasing histamine doses. n = 6 per group, *p < 0.01, unpaired Student's t-test, **p < 0.001, two-way ANOVA.

On day 1, the guinea pigs (n = 6) were administered 1 mL of an ovalbumin (OVA) solution (Chicken egg albumin grade II; 60 μg/mL; Sigma, St. Louis, MO, United States) mixed with 1 mg/mL aluminum hydroxide (J.T. Baker, NJ, United States) via subcutaneous (0.5 mg/mL) and intraperitoneal (0.5 mg/mL) routes. On day 8, antigenic sensitization in the asthma model guinea pigs was boosted by administering a nebulized booster of OVA (3 mg/mL) for 5 min, while the control group was administered nebulized physiological saline solution (PSF). On day 15, the first antigenic challenge was performed on the asthma model guinea pigs with nebulized OVA (1 mg/mL). For subsequent challenges on days 25 and 35, a concentration of nebulized OVA (0.5 mg/mL) was administered during each antigenic challenge (Figure 1A). The control group (n = 6) received PSF under the same conditions. The aerosols containing OVA or PSF were generated using a US-1 Bennett nebulizer (flow rate of 2 mL/min; Multistage Liquid Impinger, Burkard Manufacturing Co., Hertfordshire, United Kingdom), which produced particles of various sizes: <4 µm (44%), 4–10 µm (38%), and >10 µm (18%). During each antigenic challenge, the acute broncho-obstructive response to the antigen was evaluated by recording data using barometric plethysmography for freely moving animals.

### 2.3 Barometric plethysmography

Following the method described by Hamelmann et al. (1997), pulmonary function was indirectly evaluated using a whole-body single-chamber plethysmography system (Buxco Electronics Inc., NY, United States). A baseline broncho-obstruction index (BI) was obtained before the challenge and compared with the BI measured after the challenge. The barometric plethysmography system works by directly measuring pressure variations through a differential transducer connected to a preamplifier, which detects pressure changes caused by the heating and humidification of the air volume inhaled and exhaled by the guinea pig. Each BI value was averaged over a 15-second period, and the average of the last 5 min of each period was used for analysis. The detected parameters were used to calculate the BI using the following formula:

$$\text{BI}=\left[\left(\text{Te}-\text{Tr}\right)/\text{Tr}\right]\left[\text{PFE}/\text{PFI}\right]$$

Where:

Te = Total expiratory time (s).

Tr = Total relaxation time (s).

PFE = Peak expiratory flow (maximum positive pressure, cmH_2_O).

PFI = Peak inspiratory flow (maximum negative pressure, cmH_2_O).

On day 35, airway reactivity was assessed by comparing the dose-response curves obtained through the administration of increasing doses of histamine before and after the antigenic challenge with OVA (Vargas et al., 2010). Initial baseline BI values were recorded, followed by nebulization of increasing doses of histamine (0.001–0.32 mg/mL; Sigma St. Louis, United States). For each dose, the BI was recorded continuously for 5 min, and the final average was calculated. The response to histamine was determined when histamine induced bronchoconstriction that tripled the baseline BI value. Once the BI had decreased to approximately 50% of its baseline value (Bazán-Perkins et al., 2004), the third antigenic challenge with OVA was administered. Three hours after the OVA challenge, a second cumulative histamine dose-response curve was performed following the same protocol as the first curve.

### 2.4 Immunohistochemistry

Twenty-four hours after conducting the histamine dose-response curve, guinea pigs were euthanized with a lethal dose of sodium pentobarbital (28 mg/kg; PiSa, Mexico). The segment from the left lower lung lobe of guinea pigs, embedded in 10% formalin-buffered solution, were dehydrated using a dehydration series consisting of the following steps: distilled water (dH_2_O) for 10 min, 96% alcohol for 10 min, absolute alcohol for 10 min, and xylene for 10 min. Subsequently, the tissues were embedded in paraffin. Sections with a thickness of 3–4 μm were made using a microtome.

On day 1 of the immunohistochemistry protocol, the slides were deparaffinized in an incubator at 30 °C for 30 min and rehydrated using a solution of xylene for 10 min, absolute alcohol for 5 min, 96% alcohol for 5 min, and dH_2_O for 10 min. Antigen retrieval was performed with a 10 mM sodium citrate buffer at pH 6 for 10 min in a microwave oven. Non-specific sites were blocked using 2% horse serum from the Universal R.T.U. Vectastain Kit (Vector Laboratories, United States). The samples were incubated overnight at 4 °C with monoclonal antibodies against the following proteins: MYL9/MYL12a/b, MYH11, filamin 1, and transgelin. Due to concerns regarding the potential cross-reactivity of antibodies directed against actins (Dugina et al., 2009), a detailed analysis of their specificity was undertaken. According to the manufacturers, each antibody was designed to recognize a distinctive and characteristic epitope of the corresponding isoform: β-actin (ACTB), γ1-actin (ACTG1), and α-smooth muscle actin (ACTA2). For ACTA2, a monoclonal antibody targeting a specific N-terminal peptide was employed; for ACTB, an antibody directed against the C-terminal region; and for ACTG1, an antibody generated against the recombinant N-terminal region 1–17 (Table 1). To minimize the potential for cross-reactivity, computational sequence alignment analyses were performed using BLAST to compare sequence identity and differences among the three proteins. For ACTG1, the antibody was generated against amino acid residues 1–17 (UniProt ID: P63261), which differ substantially from the corresponding regions in ACTB and ACTA2, making cross-reactivity unlikely. Similarly, the ACTA2 antibody was developed against an N-terminal region (exact sequence undisclosed) that presents notable differences from the other isoforms, further reducing the probability of cross-recognition. For ACTB, the antibodies used for detection were primarily generated against C-terminal sequences; although the exact sequence is undisclosed, the likely targeted region shows considerable differences from ACTA2, also excluding cross-reactivity. Because ACTG1 shares identical regions with ACTB, cross-reactivity was experimentally evaluated using direct positive-labeling assays during immunohistochemistry, confirming antibody specificity under our experimental conditions. The slides for the blank controls were treated in the same way as the experimental samples, except for the primary antibody incubation.

**TABLE 1:** Antibodies used in this study.

Protein	Antibody	Dilution
α-actin (ACTA2)	Mouse monoclonal anti-actin, α-smooth muscle, A-2547, Sigma Inmunochemicals (Clone 1A4)	1:500
β-actin (ACTB)	Mouse monoclonal β-actin antibody, Genetex, (Clone G5512)	1:250
γ-actin (ACTG1	Mouse monoclonal γ-actin antibody, Santa Cruz Biotechnology, sc-65638 (Clone 1–17)	1:100
Filamin A (FLNA)	Mouse monoclonal filamin 1 antibody, Santa Cruz Biotechnology, sc-71118 (Clone 3fl80)	1:250
Myosin heavy chain (MYH11)	Mouse monoclonal MYH11 antibody, Santa Cruz Biotechnology, sc-6956 (Clone G-4)	1:50
Myosin light chain (MYL9)	Mouse monoclonal MYL9/MYL12 A/B antibody, Santa Cruz Biotechnology, sc-28329 (Clone E-4)	1:100
Transgelin (TLNG)	Mouse monoclonal transgelin antibody, Santa Cruz Biotechnology, sc-53932 (Clone 6g6)	1:100

On day 2 of the immunohistochemistry protocol, the samples and controls were incubated with 3% hydrogen peroxide (30% concentration, HYCEY, Mexico) and then with the secondary antibody from the Universal R.T.U. Vectastain Kit for 1 h at room temperature. Afterward, the samples and controls were washed and incubated with a peroxidase/streptavidin complex from the Universal R.T.U. Vectastain Kit. Finally, the DAB substrate kit, peroxidase, with nickel (3,3′-diaminobenzidine) was used as the chromogenic substrate. The samples and controls were counterstained with Mayer’s hematoxylin (All from Vector Laboratories, ciudad, United States) for 10 min, subjected to differentiation using a 2% glacial acetic acid solution, and incubated with a 0.03% ammonium hydroxide solution (Sigma St. Louis, EU) for blue staining. The slides were washed twice with TBS-Tween 1% at the end of each process.

To determine the expression of the positive marker of the proteins analyzed by immunohistochemistry, images were captured using an optical microscope at 10x and 40x magnification. The obtained images were then analyzed using the ImageJ-Fiji program (Open source). This involved evaluating the total number of pixels showing positive staining for each protein. Five random quadrants were selected from the images corresponding to the bronchi and tracheal smooth muscle in each guinea pig from both the allergic asthma model and control groups. Each quadrant was subjected to Color Deconvolution analysis, where pixels were separated by color depending on the technique used to reveal the antibody marker. For this analysis, the diaminobenzidine-hematoxylin function was used, as the chromogen employed in the immunohistochemistry was diaminobenzidine, and the counterstaining performed was hematoxylin. The resulting image corresponding to the DAB staining was converted to black-and-white pixels, with white pixels indicating positive staining and black pixels representing the background. A pixel histogram was then generated to analyze the two peaks corresponding to white (positive staining) and black (background), allowing for a comparison of the total pixels corresponding to the staining intensity.

### 2.5 Bronchial smooth muscle proteome-interactome

The proteins analyzed by immunohistochemistry were organized into a list within the publicly available STRING functional protein association networks program, generating a preliminary interactome based solely on protein-protein interactions. The enrichment value corresponded to the functional and biological probability percentage of interaction between the analyzed proteins. Based on these results, a new format of the interactome was constructed, incorporating the previously obtained protein expression data. The interactome displayed proteins with increased expression (red), unchanged expression (yellow), and decreased expression (blue) in the context of allergic asthma.

### 2.6 Polyacrylamide gel electrophoresis

After the euthanasia of the guinea pigs, two pools of dissected tracheal tissue, one from 6 controls and the other from 6 asthma models, were homogenized separately in phosphate buffer (6.7 mM K_2_HPO_4_ at pH 7.4 with 0.04 M KCl and 1 mM MgC_l2_, Sigma, St. Louis, MO, United States) at 4 °C. The pooled samples were sonicated three times at 30% amplitude for 30 s with 1-minute intervals on ice (Vibra-cell 75,185; Sonics and Materials Inc., Newtown, CT, United States). The suspension was then desalted and precipitated at −20 °C using a solution containing acetone, 10% trichloroacetic acid, and 20 mM dithiothreitol (DTT), all sourced from Sigma (St. Louis, MO, United States). The resulting cell pellets were resuspended in phosphate buffer at −4 °C. Protein quantification was performed using the DC Protein Assay Kit (Bio-Rad, Hercules, CA, United States). Following quantification, electrophoresis was performed.

For one-dimensional electrophoresis (1D), 12-well NuPAGE™ Bis-Tris 4%–12% gels (1.0–1.5 mm) were run under reducing conditions with 2.5% 2-mercaptoethanol, following Laemmli’s (1970) method. Proteins were loaded at various concentrations (15 μg, 30 μg, and 50 μg per lane) and separated using a commercial mini-system (Mini-Protean II) at 80 V for stacking and 120 V for separation, powered by a Power Pack 3,000. The 1D electrophoresis included the commercial molecular weight marker Precision Plus Protein All Blue, all provided by Bio-Rad (Hercules, CA, United States).

### 2.7 Two-dimensional electrophoresis

For two-dimensional (2D) electrophoresis, a hydration solution was prepared for each pooled sample, consisting of 7M urea, 2M thiourea, 4% CHAPS, 60 mM DTT, and bromophenol blue (all from Sigma, St. Louis, MO, United States), and pH 3–10 ampholytes. Each pooled sample, containing 150 μg of protein, was brought to a final volume of 125 μL and loaded onto ReadyStrip™ IPG Strips with mineral oil. The samples were allowed to hydrate at room temperature (25 °C–27 °C) for up to 16 h. The strips were then subjected to isoelectric focusing using the PROTEAN IEF Cell in three steps: 250 V linear voltage for 20 min, 4000 V linear voltage for 2 h, and 4000 V until reaching 10,000 V/h with a rapid slope, with an amperage of 50 mA per strip, using Electrode Wicks (all from Bio-Rad, Hercules, CA, United States).

After isoelectric focusing, the strips were incubated for 15 min in an equilibration solution with DTT (1.5 M Tris pH 8.8, 6M urea, 87% glycerol, 2% SDS, bromophenol Blue, 60 mM DTT), followed by 15 min in an equilibration solution with iodoacetamide (1.5 M Tris pH 8.8, 6M urea, 87% glycerol, 2% SDS, bromophenol blue, 2.5% iodoacetamide). The strips were then placed in NuPAGE™ Bis-Tris 4%–12% IPG-well gels for 1D electrophoresis as described earlier.

### 2.8 Mass spectrometry

From the 1D and 2D polyacrylamide gels, the spots and bands were manually excised using a micropipette. These were then reduced with 10 mM DTT, alkylated with 100 mM iodoacetamide, and destained with ACN: NH_4_HCO_3_ 50 mM (50:50 v/v). Protein digestion was subsequently performed with mass spectrometry-grade trypsin (Promega V528A, Wisconsin, United States) by incubation at 37 °C for 18 h. After the incubation period, the peptides were extracted with a solution of ACN: H_2_O: formic acid (50:45:5 v/v), and the sample volume was reduced in a concentrator (Eppendorf 5,301, Hamburg, Germany) and desalinated using a C18 column (ZipTipC18, Millipore, Sigma, Massachusetts, United States). The sample was then deposited in triplicate on a dedicated plate using α-cyano matrix and analyzed in a MALDI TOF/TOF 4800 mass spectrometer. Once the MS/MS spectra were obtained, a search was conducted using the Paragon algorithm from Protein Pilot software, applying a confidence threshold of 66%.

### 2.9 Statistical analysis

Histamine airway reactivity was assessed by determining the average 200% provocative dose (PD_200_), which is the interpolated histamine dose that caused a three-fold increase in the baseline BI. The change in histamine responsiveness induced by antigen challenge was evaluated using the PD_200_ ratio, defined as the PD_200_ value observed after OVA challenge divided by the PD_200_ value measured before the challenge. Significant differences between the control and allergic asthma model groups were evaluated using an unpaired Student's t-test, while obstructive differences in each antigenic challenge were assessed using a two-way ANOVA. Associations between pixel levels related to the expression of cytoskeletal proteins and changes in airway reactivity, as well as correlations between cytoskeletal protein expression levels, were assessed using Pearson’s correlation coefficient analysis. Statistical significance was determined with a two-tailed P value of <0.05.

## 3 Results

### 3.1 Allergic asthma model in Guinea pigs

Antigenic challenges with OVA induced transient increases in BI in guinea pigs sensitized with the antigen, whereas control guinea pigs challenged with PSF showed no changes. The maximum BI reached after each antigen challenge was significantly greater in guinea pigs from the asthma model compared to the controls (p < 0.001, n = 6 per group; Figure 1B). The ratio of PD_200_ to histamine after versus before the antigenic challenge was significantly lower in the asthma model guinea pigs compared to the control guinea pigs (p < 0.05, n = 6 per group; Figure 1C).

### 3.2 Localization of proteins in the smooth muscle of bronchi

**FIGURE 2:**
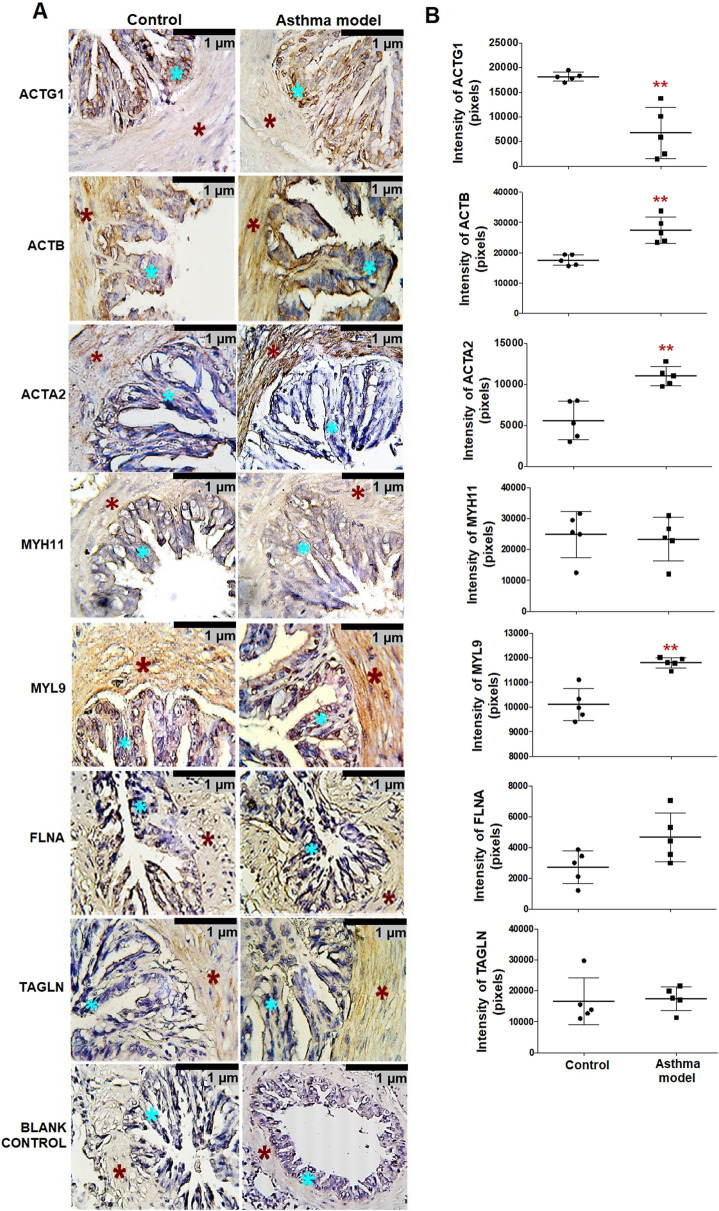
Expression and localization of proteins in bronchial smooth muscle of guinea pigs from the asthma model. **(A)** Micrographs showing the localization of positive staining for bronchi proteins in guinea pigs from both the allergic asthma model and control groups. Positive staining in the airway smooth muscle (ASM) (red asterisk) and airway epithelium (blue asterisk) in both groups. Blank control micrographs correspond to background staining controls. Chromogen: DAB; counterstained with hematoxylin. Optical microscope, 40x magnification. **(B)** Pixel expression of the positive mark for proteins analyzed by immunohistochemistry. White bars represent the control group, and black bars represent the allergic asthma model. ACTG1: γ-actin, ACTB: actin-β, ACTA2: α-smooth muscle actin, MYH11: myosin heavy chain 11, MYL9: myosin light chain, FLNA: filamin A and TAGLN: transgelin. Analysis was performed using ImageJ-Fiji software, n = 5, unpaired Student's t-test, **p < 0.01.

The localization and specific expression of proteins in bronchial tissue, such as actin-γ1 (ACTG1), cytoplasmic actin-β (ACTB), muscle actin-α (ACTA2), myosin heavy chain 11 (MYH11), myosin light chain 9 (MYL9), filamin A (FLNA), and transgelin (TAGLN), were analyzed using immunohistochemistry with specific monoclonal antibodies. All proteins were expressed in the bronchial smooth muscle (Figure 2A). In asthma model guinea pigs, ACTG1 expression significantly decreased compared to controls, while the expression levels of ACTB, ACTA2, and MYL9 significantly increased (p < 0.01, n = 5, each group; Figure 1B).

### 3.3 Interactions and relationships of proteins in bronchial smooth muscle

**FIGURE 3:**
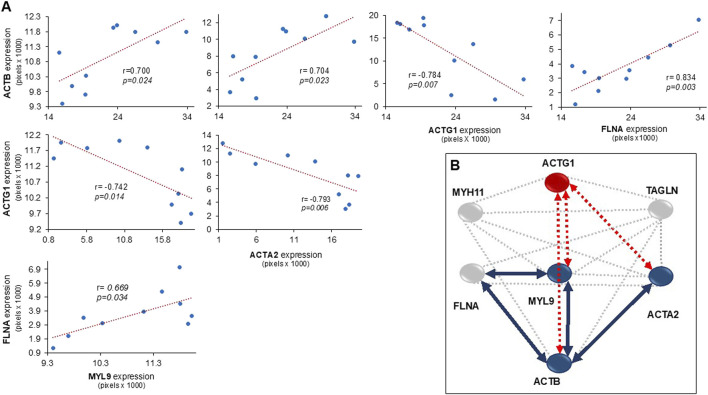
**(A)** Scatter plots illustrating the relationship between the expression levels of cytosolic proteins in bronchial smooth muscle, along with the Pearson correlation coefficients. **(B)** The interactome of proteins observed in the smooth muscle of bronchi in a guinea pig asthma model. Blue circles represent proteins with increased expression compared to control, grey circles represent proteins with no change, and red circles represent proteins with decreased expression. Blue lines indicate positive correlations between proteins, while red discontinue lines indicate negative correlations. Abbreviations are as follows: ACTG1: actin-γ1, ACTB: cytoplasmic actin-β, ACTA2: muscle actin-α, MYH11: myosin heavy chain 11, MYL9: myosin light chain 9, FLNA: filamin A, and TAGLN: transgelin. The interactome was constructed based on data obtained from the STRING: Functional Protein Association Networks program, 2023.

In the bronchi, the expression of ACTB was correlated with the levels of MYL9, ACTA2, and FLNA. Conversely, ACTG1 expression had an inverse correlation with ACTB, MYL9, and ACTA2. Additionally, the expression values of FLNA correlated with MYL9 levels (Figure 3A). Based on immunohistochemistry data, the interactomes related to the expression of MYH11, MYL9, ACTG1, ACTA2, ACTB, TAGLN, and FLNA in the bronchial smooth muscle of guinea pigs in an asthma model were generated (Figure 3B). The interactome demonstrated significant multiple interaction probability values (p < 0.05), with an enrichment value of <1.1 × 10^−6^, indicating a high level of interaction confidence. Furthermore, in this interactome, the correlation data shown in Figure 3A was included, with arrows indicating the direct and indirect correlations.

### 3.4 Relationship between AHR and proteins in bronchial smooth muscle

**FIGURE 4:**
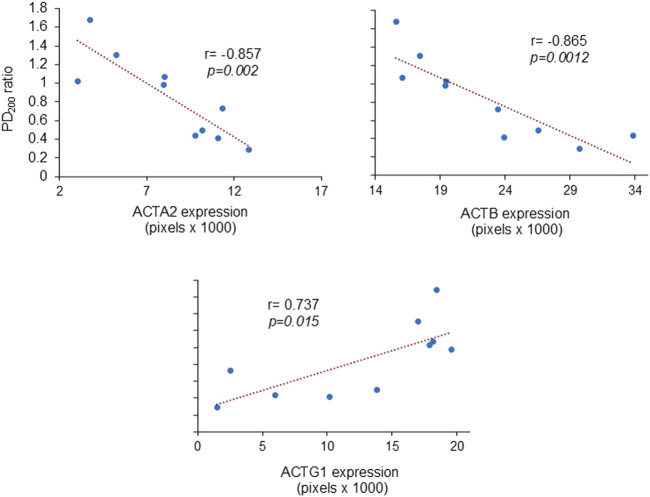
Relationship between airway hyperresponsiveness and the expression of actin isoforms in bronchial smooth muscle. Scatter plots demonstrate that the extent of the PD_200_ ratio [i.e., the change in histamine provocative dose after antigenic challenge compared to the basal (pre-challenge) value] positively correlated with the expression levels of muscle actin-α (ACTA2) and cytoplasmic actin-β (ACTB), while inversely correlating with the levels of actin-γ1 (ACTG1). The correlation coefficients are indicated by r, the Pearson correlation coefficient.

The PD_200_ ratio showed an inverse relationship with ACTA2 and ACTB levels in the bronchi, indicating that increased expression of these proteins corresponds to higher AHR. On the other hand, the PD_200_ ratio was directly associated with ACTG1, suggesting that higher AHR is linked to lower levels of ACTG1 (Figure 4).

### 3.5 Localization and identification of proteins in trachea

**FIGURE 5:**
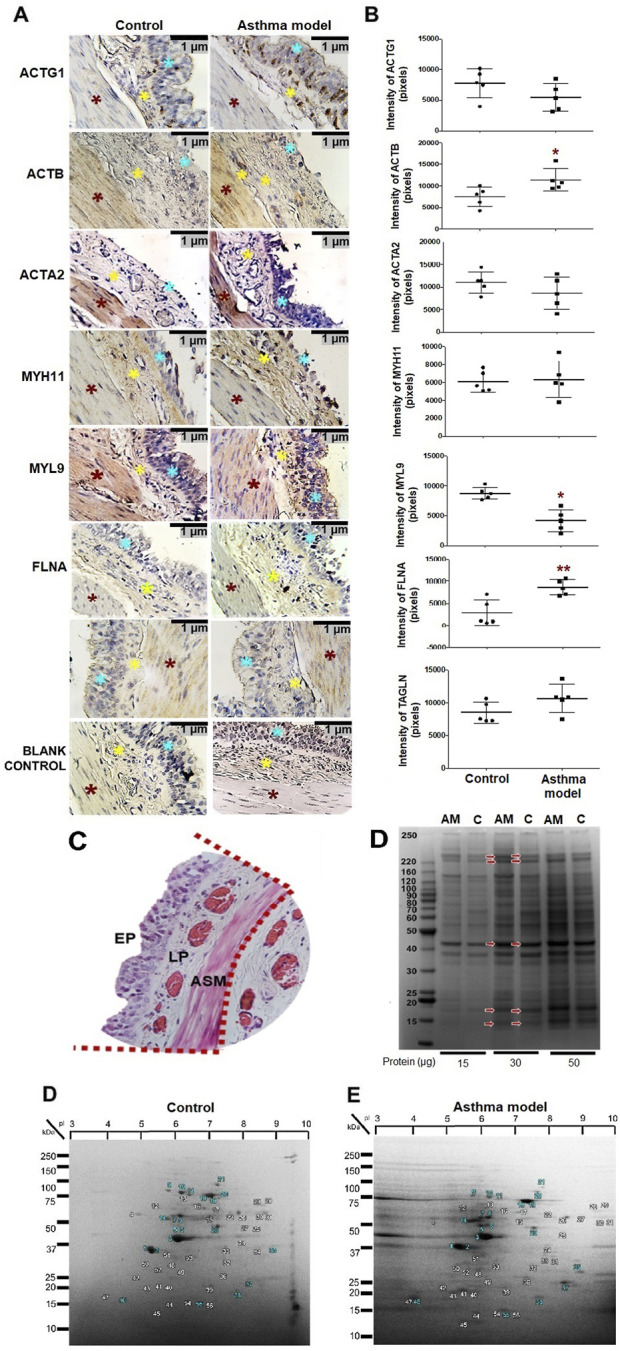
Expression and localization of proteins in tracheal smooth muscle of guinea pigs in asthma model and identification of tracheal segments, bands, and spots by one-dimensional (1D) and two-dimensional (2D) electrophoresis. **(A)** Micrographs showing the localization of positive staining for tracheal proteins in guinea pigs from both the allergic asthma model and control groups. Positive staining in the airway smooth muscle (ASM) (red asterisk), airway epithelium (blue asterisk), and lamina propria (yellow asterisk) in both groups. Chromogen: DAB; counterstained with hematoxylin. Optical microscope, ×40 magnification. **(B)** Pixel expression of the positive mark for proteins analyzed by immunohistochemistry. White bars represent the control group, and black bars represent the allergic asthma model. Analysis was performed using ImageJ-Fiji software, n = 5, unpaired Student's t-test, *p < 0.05, **p < 0.01. **(C)** Tracheal segment of isolated tissue with sections delineated by red dotted lines, corresponding to the epithelium (EP), lamina propria (LP), and ASM. **(D)** 1D electrophoresis gel. Lanes 1 and 2 (15 μg), lanes 3 and 4 (30 μg), lanes 5 and 6 (50 μg). spectrometry using the MALDI-TOF-TOF MS/MS system. SDS/PAGE polyacrylamide gel, n = 6 guinea pigs is asthma model (AM) and control **(C)** groups; image captured using ChemiDoc Imaging Systems (Bio-Rad). **(E)** Proteomic maps of guinea pigs from the control and allergic asthma model groups. A total of 56 spots were identified in both gels. The selected spots analyzed using mass microscopy are highlighted with blue numbering. Gel strips correspond to pH values of 3–10.2D SDS/PAGE gels, n = 6 guinea pigs per group; image captured using ChemiDoc Imaging Systems (Bio-Rad).

All the proteins evaluated in the bronchi were also observed in the trachea; however, the changes in expression observed in the bronchi in asthma were different in the trachea, except for ACTB, which showed increased expression in both tissues (p < 0.01 in bronchi; Figure 2A and p < 0.05 in trachea; Figures 5A,B, n = 5 each). TAGLN increased (p < 0.01) and FLNA expression decreased (p < 0.05, n = 5 each group; Figure 5A) in smooth muscle from the trachea in asthma model guinea pigs compared to controls.

The identification of proteins evaluated in guinea pigs using antibodies specific to humans, the tracheal mucosa was dissected for analysis through mass spectrometry from 1D and 2D electrophoresis gels. Tracheal tissue proteins (Figure 5C) from 6 pooled guinea pigs of the allergic asthma model and 6 pooled controls are shown in the 1D and 2D electrophoresis gels in Figures 5D,E, respectively. These gels displayed protein bands and spots with molecular weights ranging from 17–250 kDa, and for the 2D gels, spots with pH ranges from 3 to 10 (Figure 5E), present in both groups. In these gels, consistent and highly intense bands and spots, as well as some representative due to differential expression between controls and asthma models, were selected in the 1D and 2D electrophoresis, as shown in Figures 5D,E. The analysis of the selected bands and spots allowed the identification of 27 proteins in the gels from both groups based on the UniqueScore values obtained by the Paragon algorithm of the ProteinPilot software, using a confidence percentage of 66%. Finally, Table 2 lists the proteins identified exclusively in either the control group, the asthma group, or in both groups. This study confirmed the expression of peptides corresponding to ACTA2, ACTB, ACTG1, MYH11, FLNA, MYL9, and TAGLN in both control guinea pigs and the asthma model.

**TABLE 2:** Proteins identified in tracheal tissue from guinea pigs.

Only in control group	Molecular weight (kDa)
Myosin 9 (MYH9)	226.59
Peptidyl-prolyl cis-trans isomerase A (PPIA)	17.74
Only in asthma group	
Paraplegin (SPG7)	88.02/8.82
Kinase anchoring protein 3 (AKAP3)	93.29/6.12
Catalytic subunit of DNA-dependent protein kinase (DNPK1)	485/7.69
In both, asthma and control groups	
Smooth muscle actin α (ACTA2)	41.88
Cytoplasmic actin β (ACTB)	41.74
Smooth muscle cytoplasmic actin γ (ACTG1)	41.75
Myosin heavy chains (MYH11)	227.53
Calmodulin-like (CLML)	16.75
Filamin A (FLNA)	280
Myosin light chain 9 regulatory polypeptide (MYL9)	19.70/20
Transgelin (TAGLN)	22.43
SM22α transgelin (TAGLN22)	22.61

Values separated by/represent the molecular weight of peptide fragments identified by MS.

## 4 Discussion

Asthma research using animal models, such as the guinea pig, has enabled a detailed analysis of key components of the disease, including ASM contraction and AHR. Furthermore, these models allow for the evaluation of not only observable physiological features but also cellular and molecular mechanisms involved in disease severity (Álvarez-González et al., 2025). Thus, this study demonstrated that the expression of the main smooth muscle actins, namely, ACTA2, ACTB, and ACTG1, was modified in the bronchial smooth muscle—a crucial tissue in asthma (Hassoun et al., 2022)—of guinea pigs in an asthma model. Interestingly, the increase in ACTA2, but also in ACTB, was associated with the magnitude of AHR. The rise in ACTA2 expression, an actin that interacts directly with myosin filaments during contraction, has been previously observed in response to excessive ASM contraction during AHR in asthma patients, reinforcing the relevance of this protein in the disease’s pathophysiology (Slats et al., 2008). However, given that previous studies have reported stable ACTB expression as a control marker in various molecular biology techniques (Tran et al., 2006; Dekkers et al., 2007), the increased expression of ACTB was unexpected in asthma model guinea pigs. ACTB is the primary component of “cortical actin” filaments, which are arranged in submembrane networks and play a crucial role in stabilizing and anchoring actin filaments within key structures involved in cellular shortening. Furthermore, ACTB contributes to the mechanical force transmission from the actomyosin cytoskeleton to the cell membrane and extracellular matrix (Zhang and Gunst, 2008).

In line with our findings, the functional role of ACTB suggests its involvement in stabilizing and anchoring ACTA2 filaments, thereby promoting cellular rigidity and exacerbating ASM contraction—a mechanism previously proposed by Gunst and Fredberg (2003), Gunst and Zhang (2008). Although increased actin expression does not necessarily indicate greater polymerization, previous studies in canine ASM have shown that acetylcholine stimulation induces the polymerization of both ACTA2 and ACTB (Zhang et al., 2005). Our analyses revealed that the increase in ACTB expression was associated with ACTA2 expression. These findings support the hypothesis that the increased expression of these actins could be key factors in regulating AHR in asthma.

ACTG1 exhibits a differential behavior compared to ACTB and ACTA2 in the present study, as its decrease correlates with AHR in asthma. ACTB and ACTG1 share a high degree of structural similarity, differing only by four amino acid residues near the amino-terminal end (Bergeron et al., 2010). However, this minor variation, along with potential post-translational modifications, appears to confer distinct regulatory mechanisms and functions. Previous studies have reported that ACTB has a higher monomer nucleation capacity and a faster filamentogenesis rate compared to ACTG1 (Bergeron et al., 2010). Within this context, our findings suggest that ACTG1 and ACTB play interdependent roles in regulating AHR in asthma. Although the direct involvement of ACTG1 in ASM contraction remains limited, prior research on pulmonary epithelial cells has demonstrated that its depletion leads to increased ACTA2 expression and activation of Rho-associated kinase (ROCK), a key mediator of ASM contraction and AHR (Lechuga et al., 2014). It is possible that the reduction of ACTG1 in asthma may promote the upregulation of ACTA2, and probably ACTB, thereby enhancing AHR through the activation of inhibitory pathways that prevent muscle relaxation, such as those regulated by ROCK.

Since the C-terminal regions of ACTB and ACTG1 are identical, which could suggest potential cross-reactivity, we evaluated the specificity of the antibodies using immunohistochemistry. However, the signal obtained with the ACTB antibody was markedly more intense and displayed a staining pattern distinct from that of the ACTG1 antibody. This difference in both intensity and distribution indicates that, although these isoforms share high homology and some degree of cross-reactivity may be possible, the antibody specificity is sufficient to discriminate between them in the samples. If substantial or complete cross-reactivity were present, the signals would be nearly identical in both pattern and intensity, which was not observed. Therefore, these findings support the reliability of our results in accurately identifying each isoform with the selected antibodies.

MYL9, which is essential for the activation of myosin ATPase and actomyosin interaction (Sun et al., 2020), exhibited increased expression in the ASM of guinea pigs with an asthma model. However, no direct association with AHR was observed. Studies by Sun et al. (2020) have reported that the suppression of MYL9 inhibits smooth muscle contraction, and its upregulation has been documented in murine models of asthma (Sun et al., 2020; Song et al., 2023). Together with our findings, this suggests a significant role for MYL9 in ASM contraction but not in AHR. Moreover, although MYL9 did not show a correlation with ACTA2, its association with increased ACTB expression and decreased ACTG1 raises the possibility that its function extends beyond actomyosin contraction activation, potentially influencing the regulation of cytoskeletal actin isoform expression.

Key proteins involved in asthma-related contraction, such as MYH11, which forms the thick myosin filaments (Wang et al., 2021), and TAGLN and FLNA, which are involved in stabilizing actin filaments and cell membrane adhesion complexes in ASM (Iwamoto et al., 2018; Razinia et al., 2012), did not show significant changes in their expression or are related with AHR. However, FLNA expression is associated with ACTB and MYL9 expression, supporting its potential role in actin cytoskeleton remodeling (Small et al., 1986; Gerthoffer and Gunst, 2001). Additionally, FLNA has been implicated in inhibiting ASM contraction by modulating myosin ATPase activity (Méjean et al., 1992; Dabrowska et al., 1985). Thus, although FLNA did not show significant changes in expression, its role in regulating cortical actin and ASM contraction could be crucial in asthma. However, its involvement, along with TAGLN and MYH11, in the development of AHR may be limited.

Although previous sequence alignment analyses and immunohistochemistry assays indicated virtually no cross-reactivity between the ACTB and ACTG1 antibodies, a more comprehensive identification analysis using mass spectrometry was performed on tracheal smooth muscle to confirm the presence of these proteins and ensure the specificity and reliability of the results obtained. Additionally, the choice of tracheal smooth muscle for this analysis was based on the difficulty of accessing bronchial smooth muscle for isolation. The immunohistochemical expression analysis in tracheal smooth muscle, prior to mass spectrometry, revealed differences in protein expression in relationship bronchial smooth muscle. These differences between tracheal and bronchial smooth muscle had been previously reported in an equine asthma model (Matusovsky et al., 2016) where bronchial smooth muscle showed higher contraction speed and myosin ATPase activity compared to tracheal smooth muscle. These findings are particularly relevant, as numerous pharmacological studies focus on tracheal smooth muscle (Flores-Flores et al., 2019) while *in vivo* studies concentrate on the lower airways (Vargas et al., 2010). Nevertheless, mass spectrometry confirmed the presence of the proteins of interest and additionally revealed other proteins in both control guinea pigs and the asthma model, which could be subjects of future studies to assess their potential relationship with the increased ASM contraction and AHR in asthma.

An important consideration in this study is that it focused exclusively on male guinea pigs. Notably, sex-specific differences in protein expression have been observed in ASM. For instance, in rats, the presence of sex hormones in ASM alters the expression of sex hormone receptors in this tissue (Zarazúa et al., 2016). Additionally, 17β-estradiol has been shown to facilitate relaxation of human ASM by reducing intracellular Ca^2+^ levels and activating protein kinase A (PKA) (Townsend et al., 2012). The estrous cycle of female guinea pigs lasts approximately 16 days, and this cyclicity could potentially influence ASM contractility, although this has not been fully investigated. Therefore, the results obtained in this study cannot be directly extrapolated to females. When studying ASM, it is essential to interpret findings in the context of sex, as this factor may significantly affect both ASM protein expression and AHR.

Despite the numerous insights gained, several limitations of the study should be acknowledged. First, species-specific antibodies for guinea pigs were not available, and thus could not be employed. Another key limitation is the absence of human samples from both control and asthmatic patients. While some of the analyzed proteins show expression patterns similar to those observed in humans, others may differ, potentially affecting the translational applicability of our findings. Nevertheless, the results provide relevant foundation for future research. Studies incorporating human samples and more detailed mechanistic analyses will be essential to validate these findings and deepen our understanding of protein dynamics in asthma. Furthermore, the study lacks functional validation experiments, such as pharmacological modulation with activators or inhibitors, genetic manipulation of target proteins through isoform-specific siRNA knockdown, or validation using complementary techniques like Western blotting with recombinant proteins. Implementing these approaches in future studies would strengthen the evidence regarding antibody specificity and enhance result interpretation.

In conclusion, our findings provide evidence for the differential involvement of actin cytoskeletal isoforms in the regulation of ASM contraction and their association with AHR in asthma, identifying ACTB and ACTA2 as a key determinant in the disease’s pathophysiology. In contrast, the differences in expression among actins suggest a possible mechanism of differential regulation in actin polymerization and stability in asthma. Furthermore, the identification of MYL9 and FLNA as potential regulators of actin dynamics offers a novel perspective on the actin-myosin interaction and its impact on ASM contractility. Together, these findings contribute to a deeper understanding of the molecular mechanisms regulating ASM contraction and AHR in asthma, emphasizing the relevance of actins in the disease’s pathophysiological responses. They also open new research avenues and potential therapeutic targets. This integrative approach fosters new directions in asthma research, highlighting the importance of analyzing protein interactions as essential components of the molecular puzzle of asthma.

## Data Availability

The original contributions presented in the study are included in the article/supplementary material, further inquiries can be directed to the corresponding authors.
